# DIGITAL INTERVENTIONS FOR URINARY TRACT INFECTIONS PREVENTION IN PERSONS LIVING WITH DEMENTIA: A SCOPING REVIEW

**DOI:** 10.1093/geroni/igae098.3950

**Published:** 2024-12-31

**Authors:** Kuan-Ching Wu, Basia Belza, Donna Berry, Frances Lewis, Oleg Zaslavsky

**Affiliations:** University of Washington, Seattle, Washington, United States; University of Washington, Seattle, Washington, United States; University of Washington, Seattle, Washington, United States; University of Washington, Seattle, Washington, United States; University of Washington, Seattle, Washington, United States

## Abstract

People with dementia are significantly more prone to hospitalizations for urinary tract infections (UTIs) and experience more severe health outcomes compared to those without dementia. Digital interventions, such as smartphone applications, wearable devices, and telehealth platforms, show potential for transforming UTI detection, monitoring, and adherence to preventive measures. However, there is a lack of studies examining how existing digital interventions support UTI prevention in persons living with dementia. This paper reviewed various digital interventions tested in current literature for managing or preventing UTIs in this population and identified key outcomes. Utilizing the PRISMA-ScR framework, this scoping review searched five databases for studies from January 1998 to January 2024 that included digital interventions using technological devices. After screening 1800 citations, we included seven studies and evaluated three digital interventions: Technology Integrated Health Management (TIHM), a real-time locating system (RTLS), and a smart diaper system. These studies reported various outcome variables and AI algorithms for health monitoring and UTI prediction, with sensitivities up to 91%. TIHM and RTLS were highly effective in UTI detection, while the smart diaper system had lower sensitivity for urine voiding detection. Despite the prevalence of sensor-based technology and AI algorithms for early UTI detection, most interventions lack theoretical foundations and preventive strategies, highlighting the need for comprehensive approaches involving dementia caregivers. Future research should diversify its scope and integrate preventive measures guided by clinical guidelines and behavioral theories to enhance UTI management, improve quality of life, and reduce healthcare costs for persons with dementia and their caregivers.

